# The Current Role and Relevance of a Splenectomy in Immune Thrombocytopenic Purpura Patients—A Single-Center Experience

**DOI:** 10.3390/medicina61040578

**Published:** 2025-03-24

**Authors:** Cristina Ana-Maria Dan, Laurențiu Vasile Sima, Radu Gheorghe Dan, Ioana Ioniță, Octavian Marius Crețu, Gelu Mihai Brează, Alexandra Christa Sima, Claudiu Ioniță

**Affiliations:** 1 Doctoral School, “Victor Babeș” University of Medicine and Pharmacy, 30041 Timișoara, Romania; cristina.dan@umft.ro; 2 Clinic of Surgical Semiology, “Victor Babeș” University of Medicine and Pharmacy, 30041 Timișoara, Romania; sima.laurentiu@umft.ro (L.V.S.); octavian.cretu@umft.ro (O.M.C.); gelu.breaza@umft.ro (G.M.B.); claudiu.ionita@umft.ro (C.I.); 3 Center for Hepato-Biliary-Pancreatic Surgery, “Victor Babeș” University of Medicine and Pharmacy, 30041 Timișoara, Romania; 4 Hematology Clinic, “Victor Babeș” University of Medicine and Pharmacy, 30041 Timișoara, Romania; ioana.ionita@umft.ro; 5 Multidisciplinary Research Center for Malignant Hemopathies, “Victor Babeș” University of Medicine and Pharmacy, 30041 Timișoara, Romania; 6 Diabetes and Metabolic Diseases Clinic, “Victor Babeș” University of Medicine and Pharmacy, 30041 Timișoara, Romania; sima.alexandra@umft.ro

**Keywords:** immune thrombocytopenic purpura, splenectomy, complete response, partial response, non-responders, relapse, risk factors, predictive factors

## Abstract

*Background and objectives:* Immune thrombocytopenic purpura (ITP) is a rare hematological disorder characterized by an autoimmune-mediated decline in platelet count in peripheral blood. Over the years, its treatment has evolved, leading to a decline in the role of splenectomy, which was previously used as a second-line therapy. This study aims to evaluate the effects of spleen removal on the progression of the disease, regardless of the surgical procedure, by presenting a single-center experience. *Materials and Methods:* We retrospectively reviewed the medical records of all ITP patients who underwent splenectomy and were admitted to the Hematology Clinic of Timișoara Emergency City Hospital between January 1988 and June 2024. A total of 217 ITP patients who underwent splenectomy were identified over a 37-year period. Demographic data, postoperative complications, and responses to splenectomy were analyzed over a median follow-up period of 93.86 ± 104.25 months, ranging from 6 to 423 months. *Results:* Among the 217 patients included in the study, 155 (71.42%) were female and 62 (28.58%) were male, with a mean age of 38.47 ± 16.12 years. During the follow-up period, a significant decrease in the number of splenectomies was observed (*p* < 0.001). The overall morbidity rate was 14.28%. The overall response rate after splenectomy was 85.71%, with 158 patients (72.81%) achieving a complete response and 28 (12.90%) achieving a partial response. However, 28 (15.05%) of the responsive patients experienced relapse during follow-up and required additional medical therapy. Analyzing the association between comorbidities and relapse after splenectomy, the presence of diabetes (OR = 6.90, 95% CI: 2.87–16.58), hepatic diseases (OR = 64.60, 95% CI: 19.60–212.91), immune thyroid disorders (OR = 8.37, 95% CI: 2.09–33.46), and obesity (OR = 10.22, 95% CI: 3.41–30.60) were identified as risk factors for relapse using univariate analysis. *Conclusions:* Splenectomy remains the treatment with the best long-term outcomes compared to other therapies. However, concerns about early and late complications following splenectomy, along with advancements in modern ITP treatments, have led to a significant decline in the number of splenectomies performed. In univariate analyses, female gender, age over 40, and the presence of diabetes, hepatic diseases, obesity, or immune thyroid disorders were found to be risk factors for relapse following splenectomy. In the logistic regression analysis adjusted for age and sex, obesity and steatosis were significantly associated with an increased risk of relapse after splenectomy in women over 40 years of age.

## 1. Introduction

Immune thrombocytopenic purpura (ITP), formerly known as idiopathic thrombocytopenic purpura or idiopathic thrombocytopenia, is a rare hematological disorder characterized by an autoimmune-mediated decline in platelet count in peripheral blood [1]. This condition can lead to mucocutaneous bleeding, the appearance of a petechial rash, and/or bruising [2]. Over the years, its treatment has evolved, leading to a decline in the role of splenectomy, which was previously used as a second-line therapy. Although corticosteroids remain the cornerstone of initial therapy for ITP patients without contraindications [3], they are often used alongside intravenous immunoglobulin or anti-RhD immunoglobulin. However, the emergence of newer therapies, such as thrombopoietic agents, monoclonal antibodies (e.g., Rituximab), and oral spleen tyrosine kinase inhibitors (e.g., Fostamatinib), has pushed splenectomy to third- or even fourth-line treatment. Splenectomy is generally reserved for patients who fail multiple medical therapies [4].

Nonetheless, the role of splenectomy remains significant, as it is the most effective treatment for corticosteroid-resistant or relapsed ITP patients [3]. It eliminates the primary site of platelet destruction and autoantibody production [3,4]. Splenectomy achieves a complete response rate of up to 85% [5], though up to a quarter of these patients will experience a relapse within a five-year follow-up period [6].

Both open and laparoscopic splenectomy appear to have similar efficacy in terms of overall response [6], although the laparoscopic approach offers advantages such as reduced intraoperative blood loss, faster recovery, and shorter hospital stays. No statistically significant differences in postoperative complications have been observed between the two surgical methods [5,7].

Another important consideration following splenectomy is the long-term risk associated with the procedure. The risk of overwhelming bacterial sepsis in asplenic patients is well recognized [4], with the highest rate of infection occurring within the first three months post-splenectomy compared to the general population.

Although splenectomy has been extensively discussed in the medical literature over the years as part of the therapeutic arsenal for patients with refractory ITP, there are few studies that clearly identify predictive factors for the type of response or relapse in patients who respond to this treatment [8,9,10,11,12,13]. However, these data are inconsistent [12,14] and even fewer studies evaluate comorbidities as potential predictors [10,11].

The present study aims to evaluate and analyze the outcomes of splenectomy in patients with ITP, used as a second- or third-line treatment depending on the situations detailed below, who were treated in a single center and were followed up with over a long period of time and to attempt to identify potential risk or predictive factors for a specific type of response and for relapse. The demographic data of the patients, platelet counts at specific time points, the presence of splenomegaly and accessory spleens, the surgical technique used, associated comorbidities, types of therapies administered before surgery, immediate and late postoperative complications (where the retrospective nature of the study allowed their identification), the type of response after surgery, and potential relapses were analyzed.

Where certain variables appeared with high frequency, univariate analyses were conducted to assess their classification as risk factors for a specific type of response or for potential relapse. Furthermore, when certain risk factors for a specific response type or relapse were identified, we tried to perform multivariate analyses to determine potential independent predictive factors. The highly correlated variables and their large number relative to the number of recurrences made it impossible for us to interpret the statistical results in multivariate analysis.

The identification of risk factors or independent predictive factors for a particular response type or for relapse after an initial favorable response could influence the therapeutic strategy in patients with ITP and optimize splenectomy outcomes, thereby avoiding a surgical intervention burdened by immediate and long-term risks in patients for whom this therapeutic approach would most likely be unsuccessful.

## 2. Materials and Methods

We retrospectively reviewed the medical records of all patients who underwent splenectomy for ITP at the Emergency City Hospital of Timișoara between January 1988 and June 2024. This study was approved by the hospital’s Ethical Committee in February 2025. A total of 217 ITP patients who underwent splenectomy over a 37-year period were identified.

The diagnosis of ITP was established based on the presence of isolated thrombocytopenia with no other identifiable cause. Regarding the clinical presentation of patients with ITP and the severity of the hemorrhages they developed, although bleeding of varying degrees was documented in over 80% (179 out of 217 patients) at the time of diagnosis, the retrospective nature of the study, its long duration, the limited availability of data on these aspects, and the existence of multiple scales for assessing hemorrhage severity during the study period made it impossible to uniformly collect and accurately quantify these data in an objective manner. Open splenectomy was performed in all but 15 patients, who underwent laparoscopic splenectomy. Open procedures were carried out using an upper midline or left subcostal incision. Laparoscopic splenectomy was performed using the four-trocar technique, with the patient positioned in the right lateral decubitus and slight anti-Trendelenburg position.

The indications for splenectomy depended on the time at which the procedure was performed over the 37 years covered in the study, as well as on the availability of various second-line therapies. Clear indications for splenectomy included resistance or repeated relapses after glucocorticoid treatment, repeated relapses or inefficacy of second-line therapy when applied, contraindications to various second-line therapies, severe life-threatening hemorrhages, and significant impairment of quality of life due to bleeding episodes or recurrent hospitalizations.

All splenectomized patients were either corticosteroid-resistant or had relapsed following corticosteroid therapy, which was used as the first-line treatment in all cases. In 57 patients, splenectomy was used as a second or third-line therapy after the failure of glucocorticoid therapy and TPO-RAs, Rituximab and/or Fostamatinib, depending on the indication and their availability. Bone marrow examination was reserved for atypical cases or elderly patients to rule out other causes of thrombocytopenia.

To achieve optimal preoperative status, all patients received corticosteroid pulse therapy, platelet concentrates, IV immunoglobulin or thrombopoietin receptor agonists (TPO-RAs), the latter being reserved for corticosteroid-resistant patients. Platelet count was measured at the time of diagnosis, preoperatively and on postoperative days 5–7.

Among the 217 patients, 103 received a trivalent vaccine (against *Streptococcus pneumoniae*, *Neisseria meningitidis*, and *Haemophilus influenzae* type B), 14 received a bivalent vaccine (against *Streptococcus pneumoniae* and *Neisseria meningitidis*), and 8 were vaccinated only against *Streptococcus pneumoniae*. Vaccination was primarily indicated for young adults, patients over 65 years of age, immunocompromised individuals, and those with severe comorbidities. The availability of vaccines and, lastly, patient preference also influenced vaccine administration.

The response to splenectomy was defined according to previously established literature criteria:

**Complete response (CR):** Achievement and maintenance of a platelet count >100 × 10^9^/L for at least 30 days post-splenectomy, without additional treatments.

**Partial response (PR):** Achievement and maintenance of a platelet count >30 × 10^9^/L for at least 30 days post-splenectomy, with or without additional treatment.

**No response (NR):** Platelet count never exceeding 30 × 10^9^/L for 30 days or longer after splenectomy.

**Relapse (R):** A drop in platelet count below 100 × 10^9^/L for patients with a complete response, or below 30 × 10^9^/L for patients with a partial response.

The median follow-up period was 93.86 ± 104.25 months, ranging from 6 to 423 months. In all responsive patients who exhibited an increase in platelet count nearing or exceeding the upper limit, antiplatelet agents were administered in the form of acetylsalicylic acid (ASA) at a dose of 75–100 mg/day, in combination with hydroxyurea or anagrelide for persistent thrombocytosis >1500 × 10^9^/L. The treatment continued until platelet count stabilization under 600 × 10^9^/L, confirmed by two consecutive measurements during periodic checkups.

For database management and data input, we used Microsoft Excel 2016 (Microsoft Corporation Redmond, WA, USA). Statistical analyses were performed using GraphPad Prism version 9.5.1 (GraphPad Software LLC, San Diego, CA, USA). For the comparison of normally distributed data, Student’s t-test was used, while the Wilcoxon–Mann–Whitney test was applied for non-normally distributed data. For frequency comparisons, either the Chi-square test or Fisher’s exact test was used, depending on the expected frequency count. All *p*-values were two-sided and considered statistically significant at *p* < 0.05. Continuous variables, including age, follow-up period, and preoperative and postoperative platelet counts, were reported as mean ± standard deviation (SD). Univariate analyses were performed using univariate logistic regression to provide odds ratios (ORs) and confidence intervals (CIs) for assessing potential risk factors for relapse or a specific type of response following splenectomy. Kaplan–Meier curves were used to highlight the influence of different associated comorbidities on the likelihood of recurrence. Multivariate analysis using multivariate logistic regression was performed in an attempt to identify independent predictive factors for a specific type of response or for relapse after an initial favorable response. All statistical analyses were conducted with a 95% confidence interval (CI), ensuring an appropriate level of interpretative certainty.

## 3. Results

### 3.1. Baseline Characteristics

The demographics and baseline characteristics of the patients are summarized in Table 1. Among the 217 patients included in the study, 155 (71.42%) were female and 62 (28.58%) were male, with a mean age of 38.47 ± 16.12 years. The median time between ITP diagnosis and splenectomy was 21.3 months (range: 1–186.3 months). All patients included in the study were corticosteroid-resistant or had experienced multiple relapses after corticosteroid treatment, and 57 (26.26%) had received at least one additional therapy besides corticosteroid treatment prior to splenectomy. Open splenectomy was performed in 202 patients (93.08%), while the remaining 15 patients (6.92%) underwent laparoscopic splenectomy.

**Figure 1:**
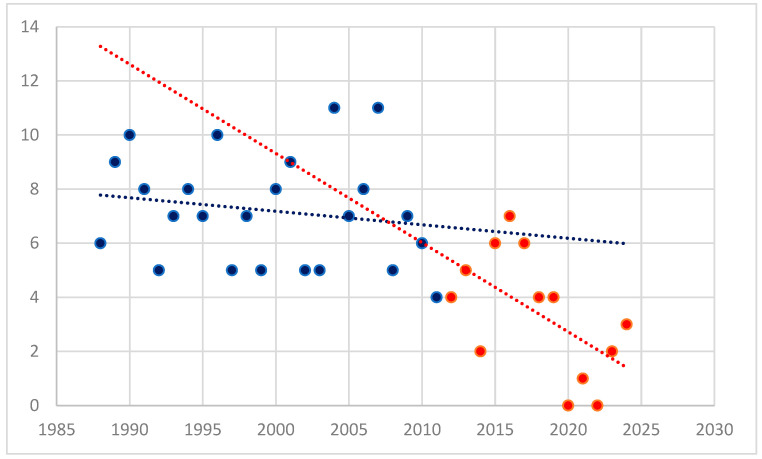
The trend in the number of splenectomies over the follow-up period; blue dots—number of splenectomies performed before 2012; red dots—number of splenectomies performed after 2012. The blue line represents the trend in the number of splenectomies up until 2012, while the red line represents the trend in the number of splenectomies after 2012.

During the follow-up period, a significant decrease in the number of splenectomies was observed (*p* < 0.001), as shown in Figure 1. The year 2012 was chosen as a landmark, as it marks the time when most second-line therapies became widely available in our Hematology Clinic.

### 3.2. Operative Outcome

The preoperative platelet count, determined after corticosteroid pulse therapy or TPO-RA administration, had a median value of 127 × 10^9^ ± 85.28 × 10^9^/L. The median postoperative platelet count, measured after splenectomy, was 470 × 10^9^ ± 420.62 × 10^9^/L, showing a significant increase (*p* < 0.001). The postoperative complications are summarized in Table 2. The median platelet count at the time of diagnosis was 28.08 × 10^9^ ± 28.60 × 10^9^/L.

The overall morbidity was 14.28%, with rates of 13.36% for open splenectomy and 26.66% for laparoscopic splenectomy, though the difference was not statistically significant (*p* = 0.053). However, in our study, laparoscopic splenectomy was associated with a significantly higher morbidity rate for early complications compared to open splenectomy (*p* = 0.021). There was no splenectomy-related mortality. Additionally, no severe infections related to splenectomy were observed during the follow-up period.

### 3.3. Response After Splenectomy

The overall response rate after splenectomy was 85.71%, with no statistically significant difference between laparoscopic and open splenectomy (*p* = 0.913). Of the 186 patients who responded to splenectomy, 158 (72.81%) achieved a complete response, while 28 (12.90%) had a partial response. However, 28 (15.05%) of the responsive patients experienced relapse during follow-up and required additional personalized medical treatment based on clinical presentation, previous therapies and response to these treatments, platelet count, and contraindications to various therapies. In the case of patients with a partial response who required adjunct therapy to support the post-splenectomy response, the treatment was also individualized based on the same criteria. None of the patients who experienced relapse were found to have secondary ITP, as these cases were not the focus of this study and were not included. All data related to the response after splenectomy are presented in Table 3.

The mean time to relapse was 23.5 ± 9.5 months, with 19 patients experiencing relapse within the first year after splenectomy. Among non-responders, a complete response was achieved in five cases (2.30%) following the removal of an accessory spleen that had not been diagnosed preoperatively or intraoperatively. Of the remaining 26 non-responsive patients, 6 experienced late spontaneous remission, which occurred more than 24 months after splenectomy while the patients were receiving medical therapy. Among the remaining 19 patients, 11 achieved stabilization of platelet counts around 30 × 10^9^/L, without requiring further aggressive therapies. The remaining eight patients experienced multiple relapses, requiring repeated therapeutic courses, moderate-to-severe bleeding episodes, and multiple hospital readmissions. During the follow-up period, 137 patients (63.13%) maintained a complete and stable response.

Analyzing the type of response based on the platelet count at the time of diagnosis or preoperatively, we did not find any statistically significant causal relationships, as shown in Table 4. Additionally, relapse does not appear to be influenced by platelet count at different time points.

The number of preoperative therapies did not influence the type of response, as summarized in Table 5.

The way in which platelet count correction was attempted as preoperative preparation, whether through pulse corticosteroid therapy or other methods, did not significantly influence the type of response or recurrence rate, as observed in Table 6. Additionally, no significant differences were observed in the preoperative platelet count between the pulse corticosteroid therapy group and the other group (126 × 10^9^ ± 84 × 10^9^/L vs. 130 × 10^9^ ± 87 × 10^9^/L, *p* = 0.76).

### 3.4. Risk and Predictive Factors for Relapse Following Splenectomy

Univariate analysis identified multiple risk factors for relapse after splenectomy, following either a complete or partial initial response, as presented in Table 7. When analyzing the association between comorbidities and relapse after splenectomy, diabetes, hepatic diseases (including toxic hepatitis, hepatitis B or hepatic steatosis), immune thyroid disorders, and obesity were found to be risk factors. The administration of at least one additional line of treatment besides corticosteroid therapy did not influence the relapse rate in our study (*p* = 0.096).

Bivariate analysis with age and sex as predictors showed that both female sex and age over 40 years are predictive factors for relapse (female: *p* = 0.031, age over 40 years: *p* = 0.002). In our study, age over 40 years had the strongest impact, increasing the risk of relapse by more than seven times.

The multivariate analysis of comorbidities was affected by collinearity and the large number of variables relative to the number of relapses, leading to instability in coefficient estimation and difficulties in interpreting statistical significance. For this reason, we conducted separate logistic regression analyses for each comorbidity, adjusting for age and sex, to assess their association with relapse. However, their statistical validity should be confirmed through larger, possibly multicentric studies. All logistic regression data are presented in Table 8.

Both for hepatitis and steatosis, Firth regression had to be applied due to perfect data separation and lack of variability, which may affect the results. Analyzing the table above, we observe that obesity is a significant predictor in women over 40 years old (*p* = 0.037).

In the case of hepatitis B, it appears to be a predictor of relapse in women under 40 years old, at the borderline of statistical significance (*p* = 0.045). However, the wide confidence interval introduces a high degree of uncertainty, most likely due to the small number of patients. For women and men over 40 years old, although the OR is very high, the extremely wide confidence intervals and *p* > 0.05 do not allow confirmation of a significant association. Steatosis appears to be a significant predictive factor in women over 40 years old.

**Figure 2:**
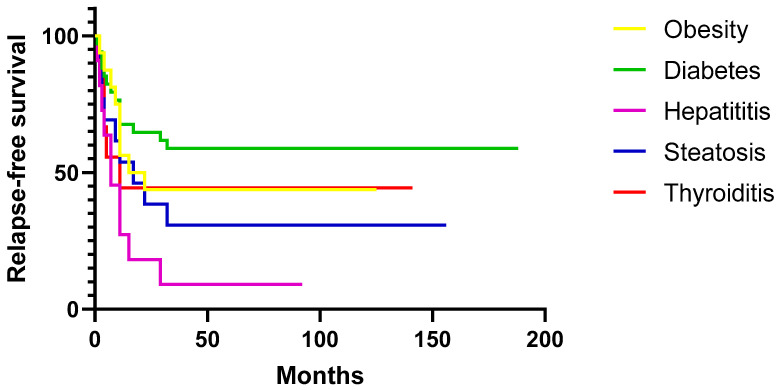
Kaplan–Meier curves—assessment of relapse risk in ITP patients with various comorbidities.

The impact of associated comorbidities on the risk of relapse in our patient cohort is illustrated in Figure 2. As can be observed, the association of liver diseases, both in the case of hepatitis and steatosis, had the greatest impact on relapse-free survival in our study.

## 4. Discussion

This study presents a single-center experience over a 37-year period. The year 2012 was chosen as a cutoff point in the studied group, as it marks the time when most second-line therapies became widely available in the clinic. This selection allows for an assessment of the current trend in splenectomy use for the treatment of ITP patients.

Although splenectomy has demonstrated its effectiveness in inducing a stable response in a significant number of patients, the choice of second-line therapy for steroid-resistant ITP remains increasingly controversial. Both patients and physicians are becoming more hesitant to opt for splenectomy [15]. This reluctance is driven, on the one hand, by the availability of effective and relatively safe medical treatments and, on the other hand, by concerns about severe early and late surgery-related complications, such as serious infections, hemorrhage, or thrombosis [16].

The aim of this study is not to compare the outcomes of open and laparoscopic splenectomy but rather to evaluate the effects of spleen removal on the progression of hematological disease, regardless of the surgical approach. Although laparoscopic splenectomy is considered the gold standard for ITP patients, it should only be performed in highly experienced and well-trained centers [4] and has been shown to have the same therapeutic efficacy as open splenectomy in terms of overall response, complications, and accessory spleen detection [7,17]. This is why we consider our study equally relevant to this objective, despite primarily discussing open splenectomies. The low number of laparoscopic splenectomies in our study is a consequence of both limited experience with the procedure and the significant decline in splenectomy cases in recent years. The slightly higher rate of certain complications after laparoscopic splenectomy observed in our study, with marginal statistical significance, can be attributed to the learning curve associated with the procedure as well as the limited number of laparoscopic interventions performed.

During the study period, a significant decrease in the number of splenectomies performed in recent years was observed. This decline is primarily due to the increased availability of medical therapies. However, it is also important to consider the impact of the COVID-19 pandemic, during which patient access to medical services across all specialties was significantly reduced. Between 2020 and 2022, only one patient underwent splenectomy, and the declining trend has persisted in subsequent years.

The effectiveness of splenectomy in terms of hematological outcomes is indisputable. Various studies report a response rate of up to 86%, with a complete response rate of 66% after a median follow-up of 92 months [4,15,17], surpassing any other treatment option. However, splenectomy should be delayed until after the first year of diagnosis due to the potential for spontaneous remission within the first year [2,18]. In our study, the overall response rate was 85.71%, with 63.13% of patients achieving a complete and stable response. Relapse after splenectomy generally occurs within the first few years, with a decreasing likelihood over time [19].

Alternative second-line therapy options include medications such as rituximab, thrombopoietin receptor agonists (TPO-RAs), and fostamatinib. However, the long-term efficacy of these treatments is lower than that of splenectomy. Rituximab, a chimeric monoclonal antibody that targets CD20, has an initial efficacy of 50–65%, but frequent relapses occur, resulting in long-term response rates of only 20–30% at 12 and 24 months [20].

TPO-RAs enhance platelet production without modulating the immune system. They are highly effective and have a low rate of adverse effects [21]. However, they are costly [20] and may require lifelong treatment, as discontinuation often leads to a decline in platelet count [22].

Fostamatinib, a newer agent that reduces macrophage-mediated platelet destruction, has shown greater efficacy as a second-line therapy rather than as a third- or later-line option. It has been associated with multiple mild adverse effects [23]. Platelet responses of ≥50,000/μL were observed in 78% of patients when used as a second-line therapy [24].

During follow-up, we identified several important risk factors for recurrence after splenectomy. Notably, female gender—particularly in patients over 40 years of age—as well as comorbidities such as diabetes, hepatic and thyroid disorders, and obesity were significant risk factors.

The effect of age on response rate and relapse following splenectomy for ITP remains controversial in the literature. While many studies suggest that younger age is advantageous for treatment success [25,26], others have found no significant correlation [17]. In our study, age over 40 was a risk factor for relapse after splenectomy, increasing it by more than seven times.

Female gender appears to influence outcomes after splenectomy, primarily due to its significantly higher incidence in women. In our study, the male–female ratio was 1:2.5. Although the *p*-value and odds ratio indicate that female gender is a risk factor for relapse after splenectomy, the statistical significance is not very strong.

There are few studies in the literature that identify obesity as a risk factor for relapse after splenectomy [11,20]. In our study, obesity was a significant risk factor for relapse (*p* < 0.001, OR = 10.22). A possible explanation for the role of obesity in post-splenectomy relapse could be its impact on immune response alterations or chronic inflammation, which may affect immune system function. Obesity appears as a predictive factor for recurrence in our study (*p* = 0.037), but its definitive confirmation, and especially its identification as an independent predictive factor, requires further studies.

Hepatic disorders like hepatitis B (*p* < 0.001, OR = 87.22) and steatosis (*p* < 0.001, OR = 18.24) were the strongest risk factor for relapse in our study. To our knowledge, there are no studies that directly evaluate the influence of hepatic disorders on recurrence after splenectomy. Liver disease may contribute to relapse through multiple mechanisms, including residual hypersplenism, altered thrombopoietin production, and liver-induced coagulopathy. In the logistic regression analysis adjusted for age and sex, hepatitis B appears to be a predictor of relapse in young women, while hepatic steatosis is associated with relapse in women over 40 years old. In the case of steatosis, the *p*-value and the relatively narrow confidence interval make the association more probable. However, for hepatitis B, causality is less likely due to the extremely wide confidence interval and a *p*-value at the threshold of statistical significance.

Thyroid disorders, particularly autoimmune thyroid diseases, were also identified as a risk factor for recurrence after splenectomy in our study. This could be attributed to immune system disruption, hormonal imbalances, or chronic inflammation. Similarly, diabetes may contribute to relapse through immunological dysfunction, chronic inflammation, or metabolic complications.

Although there are very few, if any, studies investigating the association between comorbidities and the risk of recurrence after splenectomy, the strong statistical significance found in our study could help refine patient selection for splenectomy, potentially improving overall outcomes. Large multicenter studies may be necessary to confirm these findings and allow relevant multivariate analyses to be performed, enabling the identification of independent predictive factors.

Despite the strengths of our study—including its long duration (37 years) and the ability to follow a large cohort of patients within a single center through the Hematology Clinic—it has some limitations. It is a retrospective study, and the follow-up period varied among patients, primarily due to differences in the time elapsed between surgery and our analysis. Additionally, given the extensive study period, data collection was not always comprehensive for all cases. Furthermore, as our study spans two different eras in ITP management, changes in treatment strategies over time may have influenced the results. Another aspect that may limit the statistical significance of the obtained results is the frequent association of comorbidities observed in our study.

## 5. Conclusions

Splenectomy remains a crucial component of the therapeutic arsenal for ITP patients, offering more complete and stable responses than any other treatment. However, in recent years, its use has declined, primarily due to the development of alternative therapies and concerns about serious splenectomy-related complications. Identifying predictive factors for relapse or poor response could help optimize patient selection and improve splenectomy outcomes. In our study, age and comorbidities such as obesity, diabetes, liver disease, and thyroid disorders were significant risk factors for relapse. In the logistic regression analysis adjusted for age and sex, obesity and steatosis were significantly associated with an increased risk of relapse after splenectomy in women over 40 years old. In our study, platelet count at different time points, the number of preoperatively administered therapies, and the therapy used for preoperative preparation did not influence the type of response or the relapse rate after splenectomy.

## Figures and Tables

**Table 1:** Baseline characteristics of the patients (* = statistically significant).

Variables	No. (%)	*p* Value
Age, mean (range)	38.47 (18–77)	<0.001 *
<40	81 (37.32)	
≥40	136 (62.68)	
Gender		<0.001 *
Male	62 (28.58)	
Female	155 (71.42)	
Time from diagnosis to splenectomy (mean, range)	21.3 (1–86.3) (months)	NA
Vaccination		
S. pneumoniae	125 (57.6)	NA
N. meningitidis	117 (53.91)	NA
H. influenzae	103 (47.46)	NA
Comorbidities		
Diabetes	34 (15.66)	NA
Obesity	16 (7.37)	NA
Hepatic diseases	24 (11.06)	NA
Hepatitis B	11 (5.06)	NA
Steatosis	13 (5.99)	NA
Thyroid disorders	23 (10.6)	NA
Immune	9 (4.14)	NA
Non-immune	14 (6.45)	NA
Operation	202 (93.08) 15 (6.92)	<0.001 *
Open splenectomy		
Laparoscopic splenectomy		
Accessory spleen	69 (31.79)	0.56
1	44 (20.27)	
<1	25 (11.52)	
Preoperative therapies		<0.001 *
1	160 (73.74)	
>1	57 (26.26)	
Platelet count		
Diagnosis	28.08 × 10^9^ ± 28.60 × 10^9^/L	NA
Preoperative	127 × 10^9^ ± 85.28 × 10^9^/L	<0.001 *
Postoperative	470 × 10^9^ ± 420.62 × 10^9^/L	<0.001 *
Preoperative platelet count		
With one preoperative therapy	137.37 × 10^9^/L ± 78.31 × 10^9^/L	NA
With more than one preoperative therapy	114.22 × 10^9^/L ± 96.51 × 10^9^/L	0.106

**Table 2:** Early and late complications related to open and laparoscopic splenectomy (* = statistically significant).

Splenectomy-Related Complications	Open Splenectomy (No, %)	Laparoscopic Splenectomy (No, %)	*p* Value
Early complications	18 (8.91)	4 (26.66)	0.021 *
Parietal hematoma	5 (2.47)	2 (13.33)	0.021 *
Hemoperitoneum	3 (1.48)	1 (6.66)	0.150
Subfrenic abscess	3 (1.48)	0 (0)	0.634
Pancreatic fistula	1 (0.49)	0 (0)	0.784
Epistaxis	2 (0.99)	0 (0)	0.698
Pneumonia	1 (0.49)	1 (6.66)	0.015 *
Skin rash	2 (0.99)	0 (0)	0.698
Non-infectious fever	1 (0.49)	0 (0)	0.784
Late complications	9 (4.45)	0 (0)	0.403
Peripheral thrombosis	2 (0.99)	0 (0)	0.698
Pulmonary embolism	2 (0.99)	0 (0)	0.698
Post-incisional hernia	5 (2.47)	0 (0)	0.537

**Table 3:** Response after open and laparoscopic splenectomy.

Variables	Total (%)	Open Splenectomy (%)	Laparoscopic Splenectomy (%)	*p* Value
Response				
Overall	186 (85.71)	173 (85.64)	13 (86.66)	0.913
CR	158 (72.81)	146 (73.26)	12 (80)	0.516
PR	28 (12.90)	27 (13.36)	1 (6.66)	0.455
NR	31 (14.28)	29 (14.25)	2 (13.66)	0.913
Relapse	28 (15.05)	24 (13.87)	4 (30.76)	0.100

**Table 4:** Platelet count in different types of responses.

Platelet Count	Type of Response	*p* Value
Diagnosis		
28.64 × 10^9^/L ± 28.89 × 10^9^/L	CR	NA
27.52 × 10^9^/L ± 28.31 × 10^9^/L	PR	0.85
27.38 × 10^9^/L ± 27.63 × 10^9^/L	NR	0.819
Preoperative		
130.81 × 10^9^/L ± 86.99 × 10^9^/L	CR	NA
123.19 × 10^9^/L ± 83.57 × 10^9^/L	PR	0.661
128.27 × 10^9^/L ± 86.13 × 10^9^/L	NR	0.882
Diagnosis		0.662
29.48 × 10^9^/L ± 30.60 × 10^9^/L	Relapse	
26.68 × 10^9^/L ± 26.60 × 10^9^/L	No relapse	
Preoperative		0.105
120.65 × 10^9^/L ± 91.25 × 10^9^/L	Relapse	
133.35 × 10^9^/L ± 79.31 × 10^9^/L	No relapse	
Postoperative		0.149
446.5 × 10^9^/L ± 450.06 × 10^9^/L	Relapse	
493.5 × 10^9^/L ± 391.18 × 10^9^/L	No relapse	

**Table 5:** The influence of the number of preoperative therapies on the type of response.

Type of Response (No.)	No. of Patients with 1 Therapy (%)	No. of Patients with >1 Therapy (%)	*p* Value
CR (158)	40 (25.31)	118 (74.68)	0.728
PR (28)	19 (67.85)	9 (32.14)	0.598
NR (31)	20 (64.51)	11 (35.48)	0.299

**Table 6:** The influence of the type of preoperative preparation on the response to splenectomy.

Type of Response (No.)	Group with Pulse Corticosteroid Therapy (%)	Group with Other Therapies (%)	*p* Value
CR (158)	42 (26.58)	116 (73.41)	0.52
PR (28)	11 (39.28)	17 (60.71)	0.24
NR (31)	8 (25.80)	23 (74.19)	0.93
Relapse (28)	9 (32.14)	19 (67.85)	0.78

**Table 7:** Risk factors for relapse after splenectomy for ITP patients according to univariate analysis (* = statistically significant).

Variables	Relapse Cases (%)	*p* Value	OR (CI 95%)
Gender		0.033 *	3.86 (1.11–13.38)
Male	3 (10.71)		
Female	25 (89.29)		
Age		0.002 *	6.98 (2.02–24.06)
<40 years	3 (10.71)		
≥40 years	25 (89.29)		
Female	25 (89.29)	0.004 *	6.32 (1.79–22.38)
<40 years	3 (10.71)		
≥40 years	22 (78.57)		
Male	3 (10.71)	0.369	NA
<40 years	0 (0)		
≥40 years	3 (10.71)		
Obesity	9 (32.14)	<0.001 *	10.22 (3.41–30.60)
Diabetes	14 (50)	<0.001 *	6.90 (2.87–16.58)
Thyroid disorders	9 (32.14)	0.0017 *	4.87 (1.86–12.78)
Immune	5 (17.85)	0.003 *	8.37 (2.09–33.46)
Non-immune	4 (14.28)	0.153	2.47 (0.72–8.50)
Liver diseases	19 (67.85)	<0.001 *	64.60 (19.60–212.91)
Hepatitis B	10 (35.71)	<0.001 *	87.22 (10.55–721.37)
Steatosis	9 (32.14)	<0.001 *	18.24 (5.12–64.98)
2 or more treatments prior to surgery	11 (39.28)	0.096	2.04 (0.88–4.74)

**Table 8:** Separate logistic regression analyses, adjusted for age and sex (* = statistically significant).

Variables	Relapse Cases (%)	*p* Value	OR (CI 95%)
Female	25 (89.29)	0.031 *	4.03 (1.14–14.29)
Age over 40 years	25 (89.29)	0.002 *	7.17 (2.06–24.92)
Diabetes			
Female < 40 yrs.	2 (7.14)	0.174	10.15 (0.38–272.46)
Female > 40 yrs.	10 (35.71)	0.202	2.46 (0.61–9.89)
Male > 40 yrs.	2 (7.14)	0.109	6.12 (0.67–55.53)
Obesity			
Female < 40 yrs.	0 (0)	NA	NA
Female > 40 yrs.	8 (28.57)	0.037 *	6.38 (1.12–36.28)
Male > 40 yrs.	1 (3.57)	0.274	3.97 (0.35–45.16)
Immune thyroid disorders			
Female < 40 yrs.	1 (3.57)	0.376	4.71 (0.11–209.44)
Female > 40 yrs.	4 (14.28)	0.153	2.96 (0.68–12.84)
Male > 40 yrs.	0 (0)	NA	NA
Hepatitis B			
Female < 40 yrs.	1 (3.57)	0.045 *	23.77 (1.06–532.17)
Female > 40 yrs.	8 (28.57)	0.418	3304.94 (0.000010–1.09 × 10^12^)
Male > 40 yrs.	1 (3.57)	0.473	1371.31 (0.000004–5.18 × 10^11^)
Steatosis			
Female < 40 yrs.	0 (0)	NA	NA
Female > 40 yrs.	8 (28.57)	0.0015 *	10.41 (2.44–44.35)
Male > 40 yrs.	1 (3.57)	0.106	13.09 (0.58–44.35)

## Data Availability

The datasets used and analyzed during the current study are available from the corresponding author.
